# Surgical Neonatal Sepsis in Developing Countries

**Published:** 2015-10-01

**Authors:** Ashrarur Rahman Mitul

**Affiliations:** Bangladesh Institute of Child Health and Dhaka Shishu (Children) Hospital, Dhaka, Bangladesh

**Introduction:**

Surgery on a newborn has been one of the most challenging subjects in medical science. A neonate is born with its unique physiological features of very narrow range of normalcy, beyond which it is helpless to cope with the adverse situations. Added to this burden, it has to be able to respond to life-threatening surgical conditions for its survival when these are present or do arise. Among several other factors, infections and sepsis remain as persistent and significant cause of mortality and morbidity among neonates. Newborns suffering from sepsis have a prohibitive mortality and persistent high mortality from sepsis remains as a challenge to neonatal surgical care. Survival following neonatal surgery has made a significant improvement in the developed countries because of better understanding of neonatal physiology, introduction of sophisticated devices, and availability of trained personnel and of course antibiotics treating infections effectively. But the situation still remains gloomy in the developing countries and sepsis remains as the predominant cause for high morbidity and mortality. Limitations in the developing countries are aplenty including dearth of skilled man power, scarcity of resources and facilities, unhygienic health delivery system including maternal and neonatal health, poverty, illiteracy and ignorance etc. [1-6]. Sepsis and other complications among surgical neonates have to be taken seriously into the main childhood health programs of the developing countries to be able to overcome the huge load on the overall economy of these countries. 

**The Sepsis continuum:**

Lack of uniform terminologies and definitions of sepsis and related conditions made the diagnosis and it's appropriate management very challenging worldwide. There was a need for a consensus definition of the pediatric sepsis continuum including SIRS, infection, sepsis, severe sepsis, septic shock, and multiple organ dysfunction syndrome (MODS) to aid in standardization of observational studies and evaluation of therapeutic interventions in clinical trials. The International Pediatric Sepsis Consensus Conference was held in 2002 in Texas, USA to define these issues with resource personnel from Canada, France, Netherlands, United Kingdom, and United States. Consensus definitions of SIRS, infection, sepsis, severe sepsis, shock and MODS were formulated for implementation in clinical practice among pediatric populations. Age specific values for vital signs and laboratory values were also addressed which included neonates as well. [7] 

**Sepsis and the Surgical Neonate:**

Neonates are very susceptible to bacterial and other infections. The preterm and the low birth weight neonates are even more vulnerable. Their neutrophil storage pool is diminished, chemotaxis is abnormal and cytokines and complement production is reduced; natural killer and B-cell functions are immature; type-specific immunoglobulins IgG, IgA and IgM levels are reduced. All these factors contribute to a compromised host defense. Neonatal sepsis is a systemic infection that occurs in newborns up to 28 days of age and it is a major cause of morbidity and mortality in newborns. Sepsis development can be initiated through recognition of one or more components of invading organism, including structural elements such as Gram-negative endotoxins or secreted exotoxins that stimulate the local and systemic release of endogenous inflammatory mediators. The production and release of these proinflammatory mediators can induce a systemic inflammatory response characteristic of the initial phase of sepsis. Antibiotics and supportive care remains the mainstay of treatment. [1,8] The surgical neonate has the added burdens of invasive procedures and exposure to pathogenic bacteria in the hospital environment. Although antibiotics have decreased deaths due to infection, sepsis is still a significant cause of death among the newborn surgical patient. Often, sepsis is the final common pathway to death in a critically ill neonatal patient who suffers from a postoperative complication or requires long-term mechanical ventilation and TPN. The effect of sepsis on respiratory, cardiac and renal function is evident and related directly to the pre-mortality incidents. Surgical site infections, postoperative sepsis, peritonitis, pneumonia, urinary tract infections, shunt infections, meningitis, sepsis with renal failure in posterior urethral valve and other obstructive uropathies are the different kinds of infection and sepsis related situations encountered among surgical neonates. [3, 9, 10] Surgeries involving tracheo-esophageal tract, Hepato-biliary tree, gastro-intestinal anomalies like intestinal obstructions of varied etiologies, bowel perforations, necrotising enterocolitis (NEC), anorectal malformations, anterior abdominal wall defects e.g. ruptured omphalocele or gastroschisis with evisceration where bowel is edematous and often may be necrosed, ruptured neural tube defects, musculoskeletal infections are most susceptible to develop sepsis. Most of the infective complications occur when the surgeries are performed on an emergency basis. [2, 3,11-13] Klebsiella pneumoniae, other gram-negative rods (Escherichia coli, Pseudomonas spp, Acinetobacter spp), and Staphylococcus aureus and anaerobes are the principal microbes responsible for sepsis. Occasionally infections are polymicrobial and multidrug resistant. Despite the emergence of newer broad spectrum antibiotics, no single agent has been discovered to give equally effective coverage. [14-16] Signs and symptoms are nonspecific, which makes the diagnosis difficult. The routinely used laboratory tests are not effective methods of analysis, as they are extremely nonspecific and often cause inappropriate use of antibiotics. Examples of clinical findings indicating an infection include petechiae and purpura in the setting of hemodynamic instability; fever, cough, and hypoxemia in the setting of leukocytosis and pulmonary infiltrates; or distended tympanitic abdomen with fever and leukocytosis associated with a perforated bowel. [7, 8] A rapid presumption of sepsis may be made at bedside using a "sepsis screen" that may help to guide the need of antibiotic therapy and in some cases surgical intervention. Commonly used parameters are total leukocyte count (TLC), immature to total neutrophil ratio (IT ratio), micro erythrocyte sedimentation rate (µESR) and C-reactive protein (CRP). [17]

**The scenarios in developing countries:**

Many factors are responsible for the dismal outcome of neonatal surgery in the developing countries. Sepsis and its consequences constitute the major cause of morbidity and mortality. The neonate is usually in a sorry state even when within it's mother's womb. Frequently, result of an unplanned pregnancy, there is hardly any scope for antenatal care for the mother, let alone the baby. Deliveries mostly take place outside hospital or health care facility; home delivery in a rural area, attended by traditional birth attendants is the norm. Lack of medical facilities within reach, unhygienic, unclean labour or delivery, wrapping the baby in dirty linen, cord division with unclean materials already allow organisms to gain entry. There are cultural beliefs in some societies that babies born with congenital anomalies (many are often surgical) are indicative of a bad omen for family thus defying medical treatment. Poor transportation facilities without stabilization, late referrals make the situation worse and results in delayed presentation. Preterm, low birth weight babies often do not reach the hospital. Disease processes are often far advanced when a patient reaches hospital; newborns are often referred after folk medicine or traditional remedies have failed. On many occasions sepsis has already set in by the time the baby presents to the hospital. Neonates with surgical pathology deteriorate rapidly and this necessitates prompt and adequate surgical attention that are not readily available in many of the developing countries. [2-4, 11-13] Poverty, illiteracy, ignorance are all responsible for the scenario. Even hospital-born babies in developing countries are at increased risk of neonatal infections because of poor intrapartum and postnatal infection-control practices, are 3–20 times higher than those reported for hospital-born babies in industrialized countries. Vertical transmission from mother, or by exposure to unhygienic care practices and environment are responsible. Lack of appropriate hygiene during labour and delivery, postnatal care, and feeding are major contributors; Most neonatal births and deaths in developing countries occur at home. Unfortunately, hospitals in developing countries are hotbeds of infection transmission. Intravenous catheters and ventilators if available, are introduced without sufficient attention to mitigating the substantial risk of infection they entail. When hospitals are seen as institutions where children experience poor outcomes at great cost (including enormous out-of-pocket expenses for antibiotics and prolonged length of stay), people in the communities in which they live are less likely to seek institutional care, even if advised. Whether maternal HIV infection increases the risk of hospital-acquired infections in the neonate is largely unknown. There are financial constraints, lack of trained personnel including neonatal surgeons and nurses, overcrowded and poorly ventilated environment, sharing of beds/ wards, where other septic patients are being managed, all these make already physiologically compromised surgical neonate very susceptible. Contamination of instruments, nosocomial infections are frequent. The high resistance rates are due to indiscriminate use of antibiotics.[12-15] Surgery on the newborn requires sophisticated facilities that are in general short supply. Although pediatric surgery has evolved as an independent subspecialty of surgery in many centers in developing countries, sub-specialization within pediatrics surgery is rare. Sharing wards, equipments, and nursing staffs with other surgical and even non surgical specialties are common. The pediatric surgeons do not sub-specialize in pediatric surgery; they provide general pediatric surgical care. Surgical neonates are often admitted into non-surgical wards. There is considerable stress on the available human and material resources. Inadequate human and material resources result in deferment of many elective cases and many admissions to non- surgical wards which leads to higher infective complications, increase the length of hospitalization and mortality rate. Despite the emergence of newer broad spectrum antibiotics, no single agent has been discovered to give equally effective coverage. [12-14,19, 20] Antimicrobial resistance has reached alarming levels in nurseries in developing countries including multidrug resistant bacteria. It has been estimated that 70% of pathogens isolated from bloodstream infections in hospital nurseries in developing countries may not be covered by the WHO recommended empiric regimen of ampicillin and gentamicin for neonatal sepsis. Increasing resistance to more expensive second and third line drugs is of great concern. Gross overuse of antibiotics exists in many places. More often than not, there are lack of basic hygiene, such as sinks, running water, and waste disposal facilities. Micro-organisms thrive in multi-use containers of medications, liquid soaps and other solutions, including antiseptics and disinfectants, and on inadequately reprocessed equipment. [15] Inadequacy or unavailability of NICU is a common feature in the developing countries. Surgical care is not considered an essential component of most child health programmes. Paediatric surgery has often been viewed as too expensive and as a nonessential service, and it has been excluded from most child health programmes in such countries. The most important issue surrounding the surgical care of children in developing countries is the burden of surgical diseases on pediatric populations. Epidemiological data on this subject is scarce. Surgical infections account for major contributor to morbidity and mortality. Neonates with surgical problems are especially problematic, most notably in emergency settings. Challenges for pediatric surgery including neonatal surgery in developing countries are those of definition, policy and delivery. Surgical care of children in developing countries are too expensive. Health care policy in developing countries does not reflect the surgical needs of children. It is often forgotten that surgery is an essential component of basic health care. Lack of political commitment by governments and international agencies may be the single most important reason why surgical care has not progressed in developing countries. Only a small fraction of children in developing regions have access to basic surgical care.[5]

**What went right in the developed countries? **

Mortality has marked a steady decline in the developed countries over last half century. But this improvement cannot be attributed to introduction of newer surgical techniques. Basic surgical procedures were already invented by 1960. But neonatal support system has made a huge progress which is responsible for the unique improvement. There was advancement in the following steps. (1) Widespread availability of neonatal surgeons with knowledge about neonatal surgical emergencies. 2) Parallel growth of neonatal anesthesia 3) Knowledge of neonatal physiology became well understood 4) development of sophisticated medical devices to monitor changes in neonatal physiology 5) Availability of neonatal ventilators and respiratory support system 6) Total parenteral nutrition 7) Availability of effective antibiotics and 8) Establishment of neonatal intensive care unit with trained personnel. [1]

**Future directions for the developing countries:**

Scarcity of epidemiological data prevents proper actions to be taken. Evidence based research into the difficulties faced by neonatal surgery in developing countries has to be undertaken to identify the challenges. Regional as well as international collaborations including involvement of organizations like WHO, UNICEF and other international agencies might prove fruitful. Pediatric surgery including neonatal surgery needs to be included into the national child health care policy. A comprehensive cost effective package of neonatal surgical care defined by the disease pattern of the individual countries has to be introduced. The package should address both preventive and curative sides. The protocol should identify the appropriate surgical procedures for individual diseases, give directions of criteria for referral, basic neonatal surgical screening. The care of neonates from antenatal period to delivery and onwards should be improved at the community level. Meticulous hygiene and cleanliness of whoever handling a neonate is vital. Education of the common people is must for the success of these programs. Awareness building among general population has to be undertaken. Local media, both print and electronic, has a huge positive role to play in educating people about neonatal care. A degree of training and awareness building programs for rural health caregivers, general practitioners, pediatricians and general surgeons working in rural areas may prove handy. And of course the education and training of the pediatric surgeons, nurses and ancillaries to deal with and manage neonatal surgical cases has to be undertaken. Understanding of the unique physiology of the neonates must be given emphasis. International cooperation and exchange programs between developed and the developing countries are mandatory in this regard. Training of some pediatric surgeons overseas should be arranged who themselves will be resource persons for training of others in their own country. Lessons learned from the developed placed needs to be disseminated among the pediatric surgeons dealing with neonatal surgical cases. If neonatal mortality needs to hit a lower index, neonatal surgical issues must be taken into serious consideration.

**Conclusion:**

Neonatal surgical sepsis remains as a serious obstacle to development of neonatal surgery in the developing countries. Challenges are multifactorial. But, definite decisions have to be taken to address each of them. Plans need to be worked out from the policy makers of the countries. Government as well as non-government organizations should come forward hand in hand to make these issues successful. Neonatal surgical problems should be given high priority in order to improve neonatal and infant health indices. Collaborations among the developing countries as well as with the developed world should be given importance. The need for development of evidences through research to find out current situations in individual countries cannot be overemphasized. It remains as a prime responsibility of all concerned to make this world inhabitable for each and every newborn baby, wherever on earth she/he is born.

## Footnotes

**Source of Support:** Nil

**Conflict of Interest:** None
